# Relation between Physical Fitness Components and the Occurrence and Subjective Intensity of Back Pain in Secondary School Adolescents

**DOI:** 10.3390/bs12100353

**Published:** 2022-09-23

**Authors:** Armando Cocca, Klaus Greier, Clemens Drenowatz, Nicola Lovecchio, Raúl Baños, Katharina Wirnitzer, Gerhard Ruedl

**Affiliations:** 1 Department of Sport Science, University of Innsbruck, 6020 Innsbruck, Austria; 2 Division of Physical Education, Private Educational College (KPH-ES), 6422 Stams, Austria; 3 Division of Sport, Physical Activity and Health, University of Education Upper Austria, 4020 Linz, Austria; 4 Department of Human and Social Sciences, University of Bergamo, 24129 Bergamo, Italy; 5 Faculty of Humanities and Social Sciences, University of Zaragoza, 44003 Teruel, Spain; 6 Department of Research and Development in Teacher Education, University College of Teacher Education Tyrol, 6020 Innsbruck, Austria; 7 Research Center Medical Humanities, Leopold-Franzens University of Innsbruck, 6020 Innsbruck, Austria

**Keywords:** youth, physical condition, health, lower back, cluster analysis

## Abstract

Despite the well documented benefits of physical fitness (PF) on general health, its role in back pain (BP) is still unclear. The objective of this study was to assess the association between different PF profiles and BP in a sample of adolescents. The study assessed PF and BP in a sample of 919 youth (age = 15.5 ± 1.3 years) from North and South Tyrol. A total of 531 participants (57.8%) reported no BP, whereas 4.7% (*n* = 43) reported severe BP. A two-step cluster analysis detected three main groups: the “less fit”, with significantly lower scores in all PF tests (*p* < 0.001); the “strong sprinters”, with higher speed and muscular strength than the others (*p* < 0.001); and the “flexible marathoners”, with higher flexibility and cardiorespiratory fitness (*p* < 0.001). The “flexible marathoners” showed significantly better BP scores than the “less fit” (*p* = 0.029). Cardiorespiratory fitness and trunk flexibility are potential preventive components of BP in adolescents. Since the decline in flexibility starts very early in life, it is recommended to put a particular emphasis on this component of PF. The role of other components of PF should be further investigated in the future.

## 1. Introduction

Back pain constitutes one of the most widespread health issues nowadays, affecting not only work/study efficiency but also the financial status of communities and countries [1]. For instance, in the United States, the 12-month costs related to back pain represent about 26% of the total yearly healthcare expenditure, accounting for USD 498 million [2]. Worldwide, direct expenditures for back pain represent between 0.1% and 2% of the gross domestic product, depending on the country [3]; although indirect expenses (i.e., loss of productivity) are challenging to be calculated, they are likely to be substantial due to the fact that back pain affects at least 52% of workers worldwide [3]. According to Traeger et al. [4], this issue, commonly affecting the lower (lumbar) zone of the back, is the leading cause of years lived with disability (YLD) worldwide. In line with these authors, a recent study revealed that in 2017 back pain accounted for a total of 64.9 million YLDs globally, which constitutes an increase of about 20 million YLDs compared to reports for the year 1990 [5]. The prevalence of back pain is high also in youth, and, while not common in kids aged 7 years or less, it increases with age: in early adolescence (11–13 years old) it can affect up to 18% of the population, whereas this percentage rises to over 60% already at the age of 16 [6]. A study of over 10,000 Polish youth aged 10 to 19 years old confirms that the average prevalence of back pain is about 41.5% for the entire sample, with increased chances of suffering from it at later ages [7] and peaks of up to 90% in some cohorts of adolescents [8]. The risks associated with back pain, especially when youth suffer from frequent episodes, are many: on the one side, the acute, immediate effects may range from fever, loss of balance, or general weakness to bowel dysfunction and sensorimotor deficits [9]; on the other side, youth suffering from back pain are more likely to have back pain during their adulthood, with increased risk of developing physical disabilities, such as a reduced mobility of the spine leading to lower work efficiency and quality of life [10] and to report long-term problems, such as increased feelings of anxiety and depression [11].

Among the factors that may play a role in the prevention and reduction of back pain, the previous literature points out that exercising may be paramount. In a relevant Journal of Orthopaedic & Sports Physical Therapy (JOSPT) “perspectives for patients” article, exercise is included, together with therapy and health education, as an essential component in the guidelines for health programs aiming for the prevention and treatment of back pain [12]. Furthermore, exercise seems to be as efficient as physical therapy for the improvement of back pain patients’ mindsets, particularly referring to coping skills and pain self-efficacy [13]. Exercise may additionally significantly reduce the disability associated with back pain and, when carried out at higher intensities, triggers higher exercise capacity even in the presence of back pain [14]. Finally, Cashin et al. [15] pointed out that, even during episodes of back pain, being active may be more effective than resting. In fact, these authors a variety of exercises, such as yoga, motor control activities or cardio-resistance training, are more efficient than placebo for short-term pain reduction and disability [15]. The spectrum of components of exercising related to back pain includes physical fitness (PF). PF is defined as the ability of the body systems to work together efficiently in order to perform activities. This variable is well known for its association with current and future health: for instance, it is associated with lower anxiety and higher self-esteem or improved efficiency of the cardiorespiratory system and higher bone strength [13]. Therefore, it might also contribute to the prevention and reduction of back pain. PF is composed of several elements, each of them contributing to the development and good state of the body. These elements are commonly divided into health-related (among them, cardiorespiratory fitness, muscular strength and endurance and flexibility) and skill-related (mainly referring to balance, agility and coordination) [14]. Although the previous literature has fully established PF as essential in youth [13], the findings related to back pain are contradictory. On the one side, some studies suggest that there exists a positive correlation between some components of PF and back pain: for instance, lower muscular resistance was associated with worse back health and high resistance with positive back health in a sample of adolescents from Spain [15]; in line with these findings, Semrau et al. [16] highlighted that therapies based on PF are as effective as other approaches (medical, behavioral) for the reduction of chronic non-specific low back pain. Furthermore, poor endurance seems to increase the odds of back pain occurrence in adolescents [17]. On the other side, other authors found no significant association in this regard: a study on individuals with clinically diagnosed back pain suggests that their cardiorespiratory fitness is similar to that of people not suffering from back pain and that both groups can perform fitness activities with comparable results [18]; additionally, Tanaka et al. [19] state that other variables, such as visceral adipose tissue, may have a higher significant impact on back pain than physical fitness. The lack of conclusiveness in this field is also highlighted in recent review works. For instance, a review and meta-analysis focused on the impact of muscular fitness on several health parameters pointed out that, although the former seems to improve adiposity and cardiometabolic components both in the short and long term, this cannot be confirmed for back pain [20]. In line with these findings, contradictory findings were also found by Noll et al. [21] regarding the association between flexibility, strength, endurance, aerobic capacity, balance and speed and the incidence of back pain in youth.

Considering the current state of the literature regarding the relation between PF and back pain and the fact that most studies have rather focused on single components of PF than analyzing all of them together, the aim of our study was to investigate the interaction between the components of PF with the level of back pain in a sample of adolescents; in particular, our intention was to explore whether differences in the development of said PF components may have an impact on the occurrence and intensity of back pain. We hypothesize that participants with higher PF will have lower incidence of back pain; however, this is based on the abundant knowledge on the positive contribution of all types of PA on youth health, whereas the contradictory findings from the related literature do not allow for hypotheses in this sense nor on which components of PF will have higher association with reduced occurrence of back pain.

## 2. Materials and Methods

### 2.1. Study Design

This study uses an observational, cross-sectional design with a single observation.

### 2.2. Setting

The study was carried out in 6 randomly selected secondary schools from the regions of North (4 schools) and South Tyrol (2 schools), using a simple randomization method. Schools were eligible for selection if they offered secondary level education, and they were located in the above-mentioned regions. Although considered a solid sampling method, a limitation in its use may be the lack of proportional stratification, which, in our case, could lead to selecting/excluding schools with specific characteristics (urban/rural, district, etc.). School selection and participants’ recruitment was held at the beginning of the 2018 academic year. All observations were carried out during the spring of 2018, during the regular morning physical education classes, respecting the individual academic schedule of each school involved. Tests were performed in the school gymnasiums, under similar environmental conditions and terrains.

### 2.3. Participants

The sample constituted of adolescents enrolled in grades 9 and 12 from the selected schools. Students were included in the study based on the following criteria: being regularly enrolled in one of the selected schools; participating regularly in the physical education classes; having no diagnosed physical or mental impediment for exercising; having no clinically diagnosed back pain issue. Exclusion from the final sample depended on not matching the inclusion criteria; being unable to complete all the PF tests in the battery; or not responding to the back pain item. The study protocol was approved by the Board for Ethical issues of the University (73/2021) and the school authorities of the federal state as well as the school board of each participating school. Written informed consents were collected from parents/legal guardians of the individuals included in the sample. All study procedures were in accordance with the ethical standards of the Declaration of Helsinki (2013).

### 2.4. Variables

Back pain and PF components were the observed variables of this study. Some potentially confounding factors for back pain, such as biological age and sedentary and screen time, were not included in the study. Additionally, this work did not investigate exercise behaviors of students, i.e., whether they participated in out-of-school leisure-time physical activity or whether they were sports clubs’ members. Nonetheless, the aim of this study was not to understand the source of PF but its overall current level. Finally, this study did not consider potential sex differences in relation to both back pain and PF.

### 2.5. Data Measurement

Before the measurements were carried out, some potential biases were identified. The choice of carrying out the tests at each individual school separately was taken to maintain the environmental features stable for the students as they spend most of their day in such environment and may feel safer than if asked to attend different facilities. For the same reason, their PE teachers were present during the testing, although not leading them. Tests were led by the same researchers at each of the school facilities in order to avoid biases regarding the manner in which they were carried out and to avoid differences in the feedback given to students when required. A final bias may be students’ motivation towards performing the PF tests, which may affect the results, especially in strenuous tests, such as that for assessing cardiorespiratory fitness. Although there exist quantitative methods for the estimation of participants’ effort (for instance, with the aid of heart rate monitors), they were not available in this study and such assessment was only carried out using the observation methodology.

#### 2.5.1. Anthropometric Measures

Due to the potential impact of height and body mass index (BMI) on back pain [22], anthropometric measures were taken, and BMI was calculated. The assessment of height, using a mobile stadiometer “Seca 217” (Seca, Hamburg, Germany) with an accuracy of 0.1 cm, and weight, by means of a calibrated scale “Grundig PS 2010” (Grundig AG, Neu-Isenburg, Germany) with an accuracy of 0.1 kg, were carried out with students wearing sports outfits and barefoot, in accordance with previous studies on similar populations [23,24]. Students’ BMI (kg/m^2^) was then calculated. Based on the German BMI reference system [25], they were successively categorized as anorexic, underweight, normal weight, overweight or obese.

#### 2.5.2. Back Pain

Back pain data were collected using a single item investigating the presence and intensity of the episodes with a scale with the following scores: 1 (no back pain); 2 (mild back pain); 3 (moderate back pain); 4 (strong back pain); and 5 (very strong back pain). Although not specifically validated for back pain in adolescents, a scale approach for the assessment of pain is recommended in epidemiological studies [26]. The use of a 5-choice Likert scale was also made to reflect the school grading system, which participants were familiar with. Based on the risk-intensity association for back pain proposed in previous studies [27,28,29], and in accordance with Joergensen et el. [30], based on their responses participants were grouped into three main categories: no back pain (NBP: students who stated not to suffer from back pain; score = 1); mild-to-moderate back pain (MBP: participants with low to null health risk; scores = 2–3); and severe back pain (SBP: youth whose back pain may imply higher health risk; scores > 3).

#### 2.5.3. Physical Fitness

For testing PF, participants completed the German Motor Performance Test (GMT) 6–18 [31]. The GMT is a standardized test battery consisting of 8 items as follows: 20 m sprint for the assessment of sprint velocity; balancing and walking backwards on three 3 m long beams with different width for assessing balance/coordination; jumping sideward over a middle line for 15 s to measure coordination under time constraint; stand-and-reach for trunk flexibility; 40 s push-ups and 60 s sit ups to examine muscular endurance; standing long jump for the measurement of muscular power; and 6 min run to asses cardiorespiratory fitness. The GMT 6–18 showed good scores for inter-rater reliability (0.95) and test–retest reliability (0.82), having been validated for the assessment of speed, coordination, flexibility, strength, and endurance [31]. Tests were carried out following the corresponding published GMT testing manual [31]. All tests were completed during a single session, lasting about 90 min. Tests were organized with a circuit-style organization, where smaller groups of students were distributed in the different stations and moved to the following after completion of one test. However, the 20 m sprint test was always performed at the beginning, as other tests could affect its outcomes, and the 6 min run was completed at the end of the testing session as it leads to full exhaustion and could have affected the performance in other tests if carried out before them [31]. Participants wore sports clothing and sports footwear, as required for taking part in PE classes.

### 2.6. Statistical Methods

Data are presented as means ± standard deviations. All statistical analyses were conducted using SPSS Statistics version 26 (IBM, Armonk, NY, USA). After examining the normal distribution of the data from each variable using the Kolmogorov–Smirnov test, the following step consisted in carrying out a two-step cluster analysis to identify adolescents’ PF profiles. All PF tests performed were included in the analysis as continuous variables, using the log-likelihood distance measure and the Akaike-s Information Criterion for the clustering, and adding the 3-group back pain variable in the evaluation field. A fixed number of clusters was not specified. Clustering models were hence created based on the results of the 8 test items of the GMT.

After the determination of the clusters, we carried out an initial screening of inter-cluster differences for the variable “height”, by means of one-way ANOVA with Tukey honestly significant difference (HSD) post hoc test (after confirming homogeneity of variance through Levene’s test; [32]); and for the variable “BMI group”, by means of Kruskal–Wallis test. A further ANOVA analysis followed by the Tukey HSD post hoc test was run to establish inter-cluster differences for each GMT subtest. Partial eta squared was used to assess the effect size, with values interpreted as small (values < 0.01), medium (values < 0.06) or high (values > 0.14). Finally, the Kruskall–Wallis test was used to assess differences in back pain between clusters. This test was used due to the fact that the back pain groups constitute a categorical variable with non-parametric distribution. All *p*-values were two-tailed and statistical differences were considered significant at *p* < 0.05.

## 3. Results

Descriptive sociodemographic data from the 919 adolescents (55.6% girls; mean age 15.51 ± 1.33 years; mean BMI = 21.99 ± 3.87 kg/m^2^) are provided in Table 1 below.

Descriptive results obtained from the GMT subtests are presented in Table 2. In our sample, the majority of participants (*n* = 531) stated not to suffer from back pain (57.8%), whereas 344 students (37.5%) reported mild-to-moderate back pain, and only 4.7% of them (*n* = 43) declared to have experienced severe or worse back pain. This distribution was reflected in a similar manner by sex, NBP being the most represented category in both boys and girls (girls = 58.6%; boys = 56.9%), while SBP the lowest (girls = 5.5%; boys = 3.7%).

**Figure 1:**
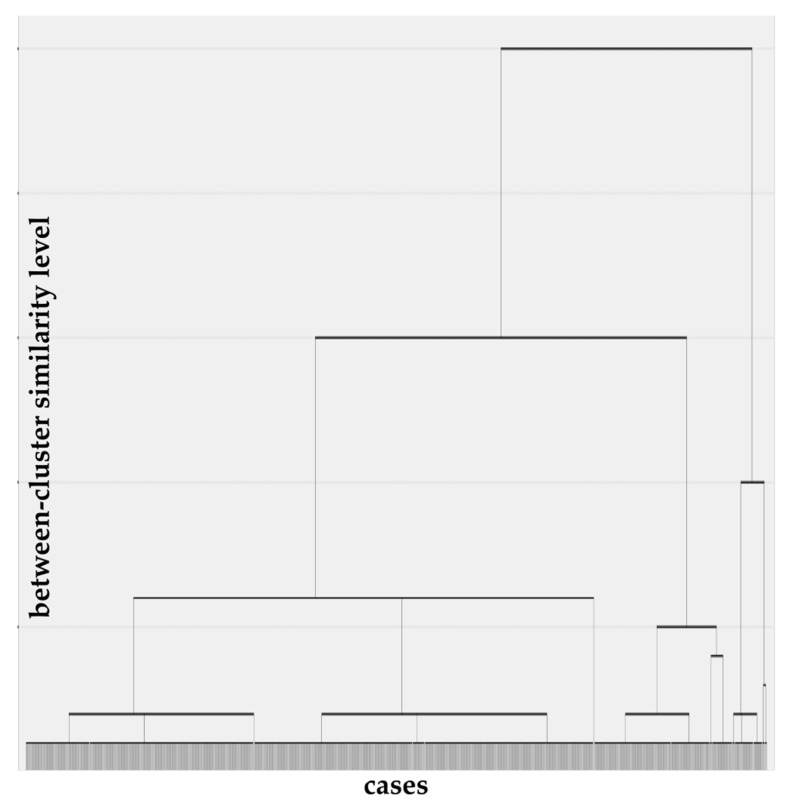
Cluster tree for the physical fitness subtests.

The two-step cluster analysis identified three distinct cluster groups based on PF characteristics (Figure 1). Of the 919 participants of the study, 15.88% (*n* = 146) were classified as “Less Fit” (LF) due to lower fitness levels than the other clusters in all eight PF tests; 38.41% (*n* = 353) as “Strong Sprinters” (SS), due to their significantly higher speed and strength performance compared to the other groups; and 45.71% (*n* = 420) as “Flexible Marathoners” (FM) due to showing significantly higher results in the 6 min running test and the flexibility test.

Contrast analyses for the cluster’s average scores in each GMT subtest showed significant differences in all subtests, with high effect size for all of them except for the trunk flexibility test, which reported medium effect size. The complete results of the analysis are shown in Table 3 below.

An ANOVA analysis (F = 116.526; df = 2; *p* < 0.001; η^2^ = 0.203) was carried out to assess differences between the three clusters regarding body height. Results showed that members of the FM cluster were significantly taller than both LF (*p* < 0.001) and SS (*p* < 0.001). No significant differences in the five categories of BMI were found across clusters (Chi square = 4.161; *p* = 0.125; df = 2). However, the following Kruskal–Wallis test (Chi square = 7.667; *p* = 0.022; df = 2) showed significant differences among clusters for back pain, the FM members presenting significantly lower prevalence than the LF ones (*p* = 0.026).

## 4. Discussion

The main objective of this study was to explore whether differences in the development of PF components may have an impact on the occurrence and intensity of back pain.

Our findings show a generally low incidence of back pain among the participants, as more than half of the sample (57.8%) stated not to have suffered from this issue. Despite this and the simultaneous extremely low rate of cases of severe back pain, more than one-third of the adolescents involved in this research pointed out having suffered from mild- to-moderate back pain episodes (37.5%). Such results are in line with those from similar research: according to Phillips et al. [6], frequency of back pain in adolescents ranges from around 20% in youth aged 11–13 years, to up to 60% at the age of 16 years. Matching these data, Kedra et al. [7] found out that back pain occurred in approximately 40% of a population of over 10,000 Polish adolescents aged 11 to 19 years old. Hence, the frequency of back pain in our sample, composed of youth within a similar age interval as in the presented studies, is within the expected range. Considering that back pain in adolescence is suggested to be most commonly associated with no particular cause, or it is due to benign factors, especially when it is moderate or less [28], our data should not suggest any risk factor in the sample of the study. However, persistent back pain, or when the intensity is severe or higher, may hint at possible detrimental consequences not only in the short term but also for individuals’ later adult life [33]. Therefore, screening and further assessing all cases of back pain is recommended in order to exclude the possibility of immediate or future problems [34]. Additionally, our data refers to the period before the outbreak of the coronavirus COVID-19 and the consequent lockdown; the study of back pain changes during the pandemic, although mostly focused on adult population, has highlighted an increase in its prevalence and intensity [35,36,37]. As it is possible that such trend affected also younger populations, the necessity of a more careful screening of back pain is even clearer.

Parallel to back pain, the less desirable PF profile, clustered as the “Less Fit” group, was not highly populated in our sample, as it consists of only 146 of the 919 participants in the study (15.88%). Studies in similar populations have shown different trends. For instance, according to Brunner et al. [38], the latest PF data in Austrian youth show steady, yet low levels overall. This is in line with the reports from the European Commission and the World Health Organization, pointing out that on average only 17% of individuals aged 11 to 17 years are sufficiently active in Austria and that the prevalence of active behaviors decreases with age from 28.2% at the age of 11 years to as low as 6.7% at the age of 17 years [39,40]. In general, differences in youth PF levels are suggested to depend on different factors, from sociodemographic (for instance, gender and age) to living environment (urban areas being a factor for lower PF compared to rural ones [41]) or participating in club sport activities [42]. As they come from a large and heterogenous population, our findings in PF differences may also depend on such factors.

Our findings further indicate that youth in the LF profile have significantly worse back pain than the FM cluster, suggesting a potential association between PF and the onset of back pain. A possible justification of this outcome may be found in the significantly different height between the two clusters. However, in our study, FM members are taller than the LF ones. This contradicts some studies hinting at the possibility that a larger height may increase the risk of suffering from back pain [22,43,44]. Nonetheless, other works emphasize that the relationship between stature and back pain is still unclear, suggesting that a role may be played by sitting height and growth rate [45,46]. Our results do not include such variables; hence, they should be taken with caution, even more as an important anthropometric factor of back pain related to stature, i.e., BMI, shows no difference across clusters. Having excluded a possible interference from these variables in the difference in back pain between LF and FM clusters may reinforce the role of PF in our sample. Yet, opposite to this, a study from Silva et al. [47] suggested that the intensity of back pain may not depend on fitness. However, the authors recognized the role of PF as determinant for reducing the occurrence of back pain episodes and their impact on the body. Moreover, the data for such study were taken from a sample of individuals with a history of chronic back pain. Therefore, the presence of at least mild-intensity back pain throughout the sample was expected. The possible impact of PF on back pain has been highlighted in a study on 92 back pain patients who underwent a series of fitness tests (6 min walking test, timed up-and-go test, sit-to-stand test, trunk flexor, extensor and side-bridge endurance tests) focused on health-related physical fitness. Results from this research emphasized the fact that patients with higher back pain and risk for central sensitization had significantly lower PF compared to the other individuals [48]. Although analyzing particular components of PF rather than all of them together, other studies suggest that low PF may lead to higher incidence of back pain. For instance, adolescents from Spain who obtained low scores in muscular resistance reported higher back pain compared to their fitter peers [15]. Poor endurance seems a factor for increased risk of suffering from back pain as well [17]. However, in general, research on the association of PF and back pain is still inconclusive [15,16,17,18,19], which may also be attributed to the overall low volume of studies, the risk of bias, and the poor quality of the evidence [21]. Despite our results brining new data in line with the body of literature supporting a positive effect of general PF on back pain, further and deeper research should be carried out in this sense.

Indeed, our study also brings some discrepant outcomes, as no difference in back pain was found between LF and SS, a cluster with higher fitness scores in all muscular strength and speed tests compared to the rest of the clusters. Speed is one of the components of PF that is not included in the so-called health-related PF (HRPF), i.e., a sub-group of PF elements with the highest positive benefits on an individual’s health [49]. In fact, differently than HRPF components, defined as those abilities that allow people to become and maintain a positive health status, speed belongs to the skill related PF, i.e., those abilities allowing people to learn new skills [50]. This may justify the fact that in our sample it seems not to affect the presence/intensity of back pain, which is among the factors having an impact on health. As per muscular strength, the previous literature presents contradictory findings. On the one hand, it seems that muscular endurance may have a protective role in reducing back pain both in terms of intensity and occurrence [51,52]. This is also supported by Smith et al. [17], whose findings highlighted that those with lower muscular endurance had higher odds of suffering from back pain, whereas high scores for this variable were associated with healthier backs in youth and supported by a study highlighting that high muscular resistance may be determining factor for avoiding back pain [15]. On the other hand, some authors point out the inconsistency of evidence regarding the relationship between muscular strength and back pain. For instance, it has been suggested that other parameters, for instance visceral adiposity, may be more important than muscular strength for the occurrence of back pain [19]. Noll et al. [21] examined several works on the topic, concluding that data on the relationship between PF components, including muscle endurance and resistance, and back pain is inconclusive. Some authors add that if in excess, muscular strength may be a leading cause of back pain in youth [53,54,55]. In support to the latter, the association between muscular imbalance (i.e., when the strength/size of muscles that are supposed to work synchronically is not well balanced) and back pain has been suggested in previous studies [56,57,58]. The fact that adolescence is a phase of growth, and that development may at times not be gradual but show sudden peaks, may contribute to asymmetric muscle growth, muscular imbalance, and consequently, higher risk of back pain. At the same time, strength training methods may also increase the risk of back problems: in fact, incorrect weightlifting techniques, excessive loads or not following the appropriate resistance training guidelines for youth may lead to increased risk of back injuries [59,60,61].

Finally, our data suggests that the main features of the FM cluster, i.e., higher cardiorespiratory fitness and higher flexibility of the trunk than both the other clusters, may be the determining factors for reduced back pain in our sample. The impact of cardiorespiratory fitness on back pain has been confirmed previously: for instance, Tataryn et al. [62] observed that general exercise and walking activities are more efficient than muscular training for reducing back pain. Furthermore, aerobic fitness and activities may prevent and reduce the effects of back pain through mechanisms such as increased blood perfusion in the area [63]. The role of aerobic exercise and cardiorespiratory fitness is further recognized in previous studies [64,65]. However, other authors have presented contrasting findings on cardiorespiratory fitness: for instance, Fernandes et al. [18] compared this variable in individuals with and without clinically diagnosed back pain, finding no significant differences and adding that the aerobic performances of both groups in different activities were comparable. A systematic review adds that data on the effect of aerobic fitness on back pain is contradictory to the date [21]. Regarding flexibility, our findings appear to confirm previous research on the topic. For instance, Kato et al. [66] found out that hamstring tightness, which plays a role in reducing trunk flexibility, is associated with higher back pain in high school athletes (mean age = 15.7 ± 0.4). This is confirmed by a study carried out by Tak et al. [67], which reports lower lumbar flexibility scores in a group of judokas with back pain compared to their peers not suffering from this issue. Sadler et al. [68] reviewed 12 articles involving a total of 5,459 participants, finding that reduced lateral flexibility and hamstring range of motion were associated with increased risk of presenting back pain. In line with these authors, a systematic review on flexibility and back pain further confirms the negative correlation between lower hamstring flexibility and higher back pain. However, the authors recommend caution due to the heterogeneity of measurement methods and potential issues with the quality of the data obtained [69], this being confirmed by a recent review that found inconclusive findings on the relation between flexibility and back pain [21].

### Limitations and Strengths

Some limitations of this study should be addressed. Firstly, we used a single-item question for the assessment of back pain. Single-item tools may have the disadvantage of being less consistent and more prone to distortion due to sociopsychological biases [70] (Bowling, 2005). In our study, this approach did not allow us to collect other important information, such as the duration of back pain or its frequency. However, single items are not uncommon in clinical research and pain assessment [26,27,71] and have been shown as effective as multidimensional tools for the evaluation of some health variables [72]. The self-reported data collection strategy for back pain may also be considered as a limitation due to the risk of non-truthfully answered questions, potential recall biases or general response biases [73]. Additionally, our study only included the assessment of PF; however, other variables may influence this variable and its relationship with back pain, among them socioeconomic factor such as, for instance, time spent in sedentary behaviors, medical history or screen time/media usage. Finally, the simple randomization sampling method may lead to particular sampling errors due to lack of stratification, for instance, the exclusion of certain types of schools based on socioeconomic or environmental (urban/rural) characteristics. Among the strengths of this study, the large sample size allows for a more accurate estimate of the studied variables and, therefore, better reflects the real condition of the specific population. Additionally, for the assessment of PF we employed the GMT, a well-known, widely used, scientific and reliable battery of tests. This ensures that the outcomes regarding the components of PF are accurate and reflective of the general fitness of the observed population.

## 5. Conclusions

The participants in our study showed a typical trend in back pain prevalence in adolescence. Lower PF, however, was associated with an increased prevalence and intensity of back pain; at the same time, our results hint at the potential beneficial role of cardiorespiratory fitness and trunk flexibility for reducing or avoiding back pain, while higher muscular strength and speed do not seem to have a significant influence on this problem. Considering the prevalence of back pain episodes already at early ages, and the risk of developing issues in the short, and mostly, long term, it is recommended to direct all effort on implementing exercise-based programs for the development of the components of health-related PF, with particular attention to flexibility, as it is known to suffer a progressive decline already from the second phase of adolescence. In this sense, physical education and school sport may be paramount, considering their proven efficacy towards health-related PF when proper pedagogical approaches, such as “Teaching Games for Understanding” or game-based fitness programs, are employed [74,75].

The relationship between PF and back pain in youth remains unclear and lacks a sufficient body of literature with high quality data. Therefore, it is recommended to further investigate this topic using state-of-the-art assessment methods, especially regarding back pain, where the use of self-reports should be replaced by objective examinations in the extent that is possible. Other variables, such as whether the participants regularly walk up/downhill or on flat terrains, the number of years after peak height velocity or biological age, could be examined in order to control for factors that may potentially contribute to the incidence of back pain among individuals. In addition, this study may serve as an initial exploratory investigation on the association of the different components of PF and back pain, and future follow-up works may focus on analyzing potential modulations of such association based on sex, biological/chronological age and in more complex variable systems involving other socioeconomic factors.

## Figures and Tables

**Table 1:** Sociodemographic data of the sample composed by 919 adolescents.

Sample (*n*)	Age	Height (m)	Weight (kg)	Body Mass Index
	Mean (SD)	Mean (SD)	Mean (SD)	Mean (SD)
Girls (511)	15.57 (1.25)	1.64 (0.06)	59.43 (11.37)	21.92 (3.81)
Boys (408)	15.44 (1.43)	1.76 (0.07)	68.79 (13.66)	22.07 (3.94)
Total (919)	15.51 (1.33)	1.69 (0.08)	63.59 (13.27)	21.99 (3.87)

**Table 2:** Descriptive results for the observed variables.

Variable	*n*	Mean	Standard Deviation
20 m speed test (seconds)	919	3.65	0.48
Beam balance (number of steps)	919	40.72	7.62
Side jump (number of jumps)	919	47.07	6.85
Trunk flexion (cm)	919	3.01	9.29
Push-ups (number in 40 s)	919	17.18	3.61
Sit-ups (number in 40 s)	919	26.23	6.51
Standing jump (cm)	919	183.27	36.22
6 min run (m)	919	1078.82	176.06

**Table 3:** Differences in physical fitness characteristics of the three clusters through one-way ANOVA with Tukey honestly significant difference post hoc test.

Test	Less Fit (LF)	Flexible Marathoners (FM)	Strong Sprinters (SS)	*p*	Partial η^2^
20 m speed test (s) *^,†,‡^	4.19 ± 0.81	3.75 ± 0.24	3.32 ± 0.22	<0.001	0.387
Beam balance (steps) *^,†^	32.53 ± 9.78	42.08 ± 5.78	42.44 ± 6.29	<0.001	0.217
Side jump (jumps) *^,†,‡^	40.39 ± 5.30	45.79 ± 5.54	51.34 ± 5.97	<0.001	0.316
Trunk flexion (cm) *^,‡^	−0.28 ± 9.59	5.72 ± 8.76	1.13 ± 8.92	<0.001	0.074
Push-ups (number in 40 s) *^,†,‡^	13.56 ± 3.58	16.44 ± 2.56	19.54 ± 3.03	<0.001	0.343
Sit-ups (number in 40 s) *^,†,‡^	19.45 ± 5.55	24.31 ± 4.12	31.28 ± 5.37	<0.001	0.442
Standing jump (cm) *^,†,‡^	144.53 ± 30.05	168.80 ± 20.55	216.34 ± 24.15	<0.001	0.574
6-min run (m) *^,†, ‡^	897.63 ± 157.66	1227.83 ± 127.84	1015.69 ± 107.20	<0.001	0.502

Note. * Significant difference between LF and FM; ^†^ Significant differences between LF and SS; ^‡^ Significant differences between FM and SS.

## Data Availability

The data presented in this study are available on request from the corresponding author. The data are not publicly available due to privacy policy restrictions.
